# Menstrual Psychosis in a Patient With Premenstrual Dysphoric Disorder Repeatedly Triggered by Minimal Alcohol Consumption: A Case Report

**DOI:** 10.1155/crps/4861191

**Published:** 2026-07-20

**Authors:** Haruka Tagusari, Kohei Nishimura, Masashi Mizutani, Kumi Moriwaki

**Affiliations:** ^1^ Kanagawa Psychiatric Center, 2-5-1, Serigaya, Konan Ward, Yokohama City 233–0006, Kanagawa, Japan; ^2^ Department of Neuropsychiatry, The University of Tokyo, 7-3-1, Hongou, Bunkyou Ward, Tokyo 113–8655, Japan, u-tokyo.ac.jp

**Keywords:** alcohol-triggered psychosis, hormonal treatment, menstrual psychosis, premenstrual dysphoric disorder

## Abstract

Menstrual psychosis, a condition characterized by acute onset, brief duration with full recovery, and psychotic or manic symptoms aligned with the menstrual cycle, has been described in the literature. However, it is not recognized as an independent diagnostic entity in either the International Classification of Diseases, 11th Revision (ICD‐11) or the Diagnostic and Statistical Manual of Mental Disorders, 5th Edition (DSM‐5), and treatment options and comorbidities associated with this condition remain poorly understood. We report the case of a 26‐year‐old woman who repeatedly developed acute psychotic symptoms, including catatonia, which was in synchrony with her menstrual cycle, with the first two episodes triggered by minimal alcohol intake. Retrospective assessment revealed the presence of premenstrual dysphoric disorder (PMDD) as a comorbidity. Although antipsychotic treatment initially improved her symptoms, relapses occurred until low‐dose estrogen–progestin (LEP) therapy was introduced, resulting in sustained remission. This case highlights the potential efficacy of hormonal intervention, the overlap between menstrual psychosis and PMDD, and the possible role of alcohol as a trigger. It emphasizes the need for a rigorous approach to diagnosis and the development of evidence‐based treatment strategies.

## 1. Introduction

Despite growing awareness of reproductive health impacts in psychiatry, menstrual psychosis, a condition characterized by acute onset, brief duration with full recovery, and psychotic or manic symptoms aligned with the menstrual cycle, remains poorly understood [1]. Although it has been reported in the literature, menstrual psychosis is not recognized as a distinct diagnosis in either the International Classification of Diseases, 11th Revision (ICD‐11) or the Diagnostic and Statistical Manual of Mental Disorders, 5th Edition (DSM‐5), and no standard treatment options exist [2–4]. Premenstrual dysphoric disorder (PMDD), included in both classifications, affects ~3%–8% of menstruating women [2, 4, 5]. PMDD is associated with an increased risk of major depressive and anxiety disorders; it also places a considerable burden on the quality of life of reproductive‐age women [6, 7]. However, the potential overlap between PMDD and menstrual psychosis has received little attention despite its possible implications for diagnosis and treatment [1, 6]. Herein, we report the case of a nulligravid woman in her mid‐twenties who repeatedly developed acute psychosis in rhythm with her menstrual cycle and was successfully treated with antipsychotics and low‐dose estrogen–progestin (LEP) therapy. Retrospective assessment indicated the presence of PMDD as a comorbidity. The first two psychotic episodes were triggered by minimal alcohol intake. This case provides insight into potential hormonal interventions for menstrual psychosis and the possible association between menstrual psychosis and PMDD, highlighting the importance of multidisciplinary management, including collaboration with gynecologists.

## 2. Case Presentation

A 26‐year‐old woman was admitted involuntarily for acute psychotic symptoms that emerged immediately after consuming two sour cocktails at a pub. Her symptoms included hallucinations, thought blocking, and insomnia, as well as sexually themed delusions in which she believed she had intercourse with a celebrity. She exhibited severe confusion and disorientation to time and place. The physical examination, including a neurological examination, was unremarkable. No abnormalities were found in blood tests, urine drug screening, head computed tomography, or electroencephalography results. The blood ethanol concentration was below the detection limit. She reported no specific dietary habits. Her past medical history—including any history of alcohol or substance abuse or dependence—was unremarkable, and she was not taking any regular medications or nutritional supplements. She was an occasional social drinker and had no history of harmful alcohol use. Her family history of mental illness included her mother’s postpartum depression and the loss of another relative to suicide. Her symptoms completely resolved within 1 week with risperidone administration (2 mg/day) and coincided with the onset of menstruation. She was discharged without any maintenance medication. She maintained a baseline mental status during outpatient follow‐up for more than a year.

However, 18 months later, a similar episode, featuring catatonic symptoms and sexually themed delusions in which she believed that she had aborted babies, occurred following the consumption of 350 mL of beer. Recovery followed risperidone (2 mg/day) and lorazepam (1.5 mg/day) treatment and coincided with the onset of menstruation. Lorazepam was discontinued after the improvement of catatonic symptoms. Following the second episode, we noted that the initial and second episodes of psychosis both had emerged about 1 week prior to menstruation, following alcohol consumption, and had resolved around the onset of menstruation. Furthermore, a retrospective assessment revealed a 2‐year history of premenstrual mood instability, irritability, and aggression, preceding the onset of psychotic symptoms and suggesting a diagnosis of PMDD. Since the hospital was a psychiatric‐only facility, the patient was referred to an external gynecology clinic during hospitalization for examination and evaluation of the potential use of LEP. Transvaginal ultrasonography and relevant laboratory tests revealed no organic abnormalities. She was discharged with aripiprazole (6 mg/day), which replaced risperidone (2 mg/day) because of possible drug‐induced parkinsonism. LEP therapy was planned in addition to aripiprazole.

However, the following month, prior to the initiation of LEP, a third episode involving catatonia occurred without alcohol intake. Although the episode resolved spontaneously with menstruation, mild symptoms recurred the following month while the patient was taking brexpiprazole (2 mg/day). In the subsequent month, concurrent administration of LEP and brexpiprazole (2 mg/day) resulted in significant improvement in both psychotic and PMDD symptoms. After discharge, in accordance with the patient’s preference and in consultation with the gynecologist, we minimized the number of menstrual cycles to once every 3 months using LEP therapy. Her symptoms remitted for over a year, with only minimal functional auditory hallucinations persisting during the premenstrual period, which caused no significant distress or impairment in daily functioning.

## 3. Discussion

The patient presented with acute‐onset psychotic symptoms that were temporally related to her menstrual cycle and remitted within a short period. The initial episode showed complete recovery for more than a year; however, after two relapses, auditory hallucinations persisted exclusively during the premenstrual period. Although menstrual psychosis is not formally recognized as a distinct diagnostic entity in current classification systems such as DSM‐5 and ICD‐11, the clinical characteristics of our case were consistent with the concept of menstrual psychosis by Brockington [1–4].

The present case suggests three important points: first, the potential effectiveness of LEP in treating menstrual psychosis; second, a possible link between menstrual psychosis and PMDD; and third, alcohol as a conceivable risk factor triggering menstrual psychosis in patients with PMDD.

Under existing diagnostic frameworks, cases such as the present one could be categorized as bipolar disorder or acute and transient psychotic disorders. Accordingly, the initial management of this case involved antipsychotic treatment exclusively for acute and transient psychotic disorders. However, psychotic symptoms relapsed despite antipsychotic therapy, whereas LEP augmentation effectively prevented recurrence. A few studies have indicated that antipsychotic medication has limited efficacy in preventing psychotic episodes, whereas others have reported successful treatment with progesterone or estrogen [1, 8, 9]. The present case highlights that menstrual psychosis may require a management approach different from typical psychotic disorders, emphasizing the need for a rigorous approach to diagnosis and the establishment of evidence‐based treatment strategies. While LEP was effective in this patient, its long‐term use should be carefully managed using a comprehensive, multidisciplinary framework involving gynecologists while considering the potential side effects, such as breast cancer or thrombosis, as well as the patient’s reproductive intentions [10, 11].

Second, the association between menstrual psychosis and PMDD remains poorly understood. Although a small number of reports describe patients with both PMDD and psychosis, menstrual psychosis itself is a rare and underrecognized condition. To the best of our knowledge, only one previous case of menstrual psychosis with PMDD has been reported: the case of a 28‐year‐old woman with PMDD who developed catatonic symptoms consistent with menstrual psychosis [12]. She was initially treated with olanzapine (5 mg/day) and lorazepam (2 mg/day); she subsequently maintained remission only with fluoxetine (20 mg/day) administered during the luteal phase. Fluoxetine, a selective serotonin reuptake inhibitor, is recognized as a first‐line treatment for PMDD [13]. Given that fluctuations in ovarian hormone levels play a central role in PMDD, these hormonal changes may also contribute to the onset or exacerbation of menstrual psychosis [6]. Although the neurobiological mechanisms underpinning menstrual psychosis remain unclear, estrogen withdrawal has been postulated to affect the central nervous system in women who are biologically more susceptible to hormonal fluctuations [14]. Moreover, estrogen may exert a protective effect against psychotic symptoms by downregulating dopamine transmission in a similar manner to the effects of antipsychotic medications [15]. Considering the high prevalence of PMDD among menstruating women, further investigation into its potential association with menstrual psychosis is warranted.

Lastly, in our case, the psychotic symptoms developed shortly after minimal alcohol intake during the first two episodes. To the best of our knowledge, an association between alcohol intake and menstrual psychosis has never been reported. However, a meta‐analysis reported that alcohol intake increases the risk of premenstrual syndrome (PMS), possibly because alcohol alters the levels of sex steroid hormones and gonadotropins during the menstrual cycle [16]. As PMDD is considered a severe form of PMS, alcohol‐induced exacerbation may contribute to the development of menstrual psychosis, as observed in this case.

In conclusion, this case of menstrual psychosis in a patient with PMDD underscores the importance of assessing menstrual cycles, as hormonal treatment may be required in the management of reproductive‐age women presenting with acute psychosis. Given that menstrual psychosis is not currently recognized as an independent diagnostic entity and no standard treatment exists, it is clinically and ethically important to engage in comprehensive, multidisciplinary deliberation, including close collaboration with gynecologists and consideration of the patient’s reproductive health. Furthermore, this case suggests a potentially underrecognized link between PMDD and psychotic symptoms, highlighting the need for further research.

## Author Contributions

Haruka Tagusari conducted the patient interview and treated the patient, performed the literature review, and drafted the initial manuscript. Kohei Nishimura and Kumi Moriwaki contributed to patient care, assisted with the literature review, and provided feedback on earlier drafts. Masashi Mizutani revised the initial manuscript.

## Funding

This research received no external funding.

## Disclosure

All authors reviewed and approved the final version of the manuscript.

## Ethics Statement

This case report was conducted in accordance with the Declaration of Helsinki. According to the institutional policy of the Kanagawa Psychiatric Center, ethical approval was not required for the publication of a single case report. Written informed consent for the publication of this case report and accompanying clinical information was obtained from the patient. All potentially identifiable information has been removed to protect patient privacy. This case report was prepared in accordance with the CARE guidelines (for CAse REports).

## Conflicts of Interest

The authors declare no conflicts of interest.

## Data Availability

The data supporting this case report are not available due to patient confidentiality considerations and to protect patient anonymity.
